# Prioritization of polygenic combinations for LOAD mouse models from ensemble machine learning on cross‐species transcriptomic signatures

**DOI:** 10.1002/alz.091329

**Published:** 2025-01-03

**Authors:** Gregory A. Cary, Ravi S Pandey, Asli Uyar, Catrina Spruce, Annat Haber, Gregory W Carter, The IU‐JAX‐PITT MODEL‐AD Center

**Affiliations:** ^1^ The Jackson Laboratory, Bar Harbor, ME USA; ^2^ The Jackson Laboratory for Genomic Medicine, Farmington, CT USA

## Abstract

**Background:**

The genetic etiology of late‐onset Alzheimer’s disease (LOAD) is complex, with over 75 identified loci contributing to disease risk. Recent efforts of the MODEL‐AD consortia have yielded several dozen mouse strains harboring variation designed to model LOAD risk alleles. Given the complex genetic architecture of LOAD, developing animal models that combine multiple risk alleles is likely essential to improving the fidelity of these models to human disease. However, it is not clear which combinations of risk alleles are optimal for modeling disease features in mice. In this study we utilize transcriptome data from existing strains to prioritize which variants should be combined in future model development.

**Method:**

We estimated the effects of genetic variants and environmental perturbations on gene expression using multiple linear regression. The computed gene‐level effects were used as features in separate regression models fit to changes in human transcriptomes from AMP‐AD cohorts and previously described disease subtypes. To mitigate multicollinearity and allow for non‐linear relationships, we employed both regularized linear models (LASSO, SVM) and non‐linear models (Random Forest, Neural Network). Variable importance was extracted from the fit models for each regression analysis. We evaluated the importance of each feature (e.g. genetic variant) across models in an ensemble approach.

**Result:**

The top mouse features prioritized by the different regression models were largely similar. Moreover, similar features were also prioritized across the different human cohorts. The top mouse features included a humanized Aβ and variants in SPI1, ABCA7, Epha1, and Sorl1. Intriguingly there were distinct sets of strains prioritized for different disease sub‐types. Looking across cohorts there were generally a typical (i.e. inflammatory) sub‐type and a non‐inflammatory sub‐type. The inflammatory subtypes prioritized similar features to the overall cohorts, while the priorities for non‐inflmmatory subtypes included Cd2ap, Shc2, and Mtmr4.

**Conclusion:**

We identified consistent predictions regarding which mouse factors are most influential in modeling disease across model classes and human cohorts. Ensembling the models was a useful approach for feature selection. These priorities informed the selection of mouse strains to breed in the development of polygenic LOAD mouse models by the IU‐JAX‐PITT MODEL‐AD center.

